# Measuring Molecular Forces With Atomic Force Microscopy 1: Solvent Influence on Hydrophobic Interactions

**DOI:** 10.1002/jemt.70111

**Published:** 2025-12-19

**Authors:** Luis N. Ponce‐Gonzalez, José L. Toca‐Herrera

**Affiliations:** ^1^ BOKU University Institute of Biophysics Vienna Austria

**Keywords:** atomic force microscopy, chemical vapor deposition, DMSO, fluorocarbon, gold, hydrophobic interactions, van der Waals forces

## Abstract

Molecular forces drive phenomena such as self‐assembly, aggregation, and protein folding, where hydrophobic interactions are paramount. However, the origin of the hydrophobic mechanism remains unknown. Advances in techniques like atomic force microscopy (AFM) have improved our ability to study this topic. Hydrophobic interactions are stronger and longer ranged than van der Waals (vdW) forces, potentially arising from water structuring, polarization, and entropic effects. In this primer, fluorocarbon surfaces were prepared via chemical vapor deposition (CVD) on gold to explore the impact of water:DMSO solvent binary mixtures on hydrophobic interactions. Force–distance curves measured with AFM were fitted to an extended vdW model, disclosing the influence of the medium polarity on the interactions.

## Highlights

- This primer outlines a method for functionalizing gold surfaces with fluorocarbon.
- Atomic force microscopy is used to evaluate the magnitude and range of hydrophobic interactions and van der Waals forces in water:DMSO mixtures between fluorocarbon surfaces.
- The data is analyzed using an extended van der Waals model for accurate interpretation.

## Graphical abstract

Atomic force microscopy enables precise evaluation of the magnitude and range of hydrophobic and van der Waals interactions. This primer presents a protocol for fluorocarbon functionalization of gold surfaces and examines how water: DMSO mixtures affect hydrophobic forces, with data interpreted through an extended van der Waals model.

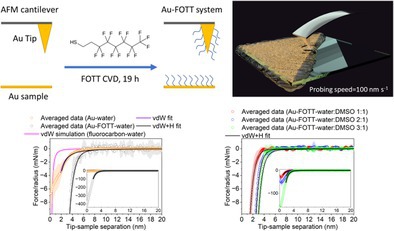

## 1 Introduction

Hydrophobic interactions are present in several physical and biological phenomena such as micellization, aggregation, protein folding, and cell‐adhesion, making them a key area of study in molecular, interface, and colloid science (Evans and Wennerström 1999; Israelachvili 2011a, 2011b). These interactions also control many technological processes. For instance, hydrophobic interactions have been applied in chromatography (van Oss et al. 1979; Srinivasan and Ruckenstein 1980), nanomaterial self‐assembly (Sánchez‐Iglesias et al. 2012), 2D material wettability (Snapp et al. 2020), catalytic reaction enhancement (Leonova et al. 2023), electrode performance (Pennathur et al. 2023), organic synthesis (Lipshutz et al. 2018), water treatment (Goswami et al. 2024; Liu et al. 2024), drug and gene delivery (Xiao et al. 2020; Falanga et al. 2024), nanocar racing (Chen et al. 2016), and so forth.

Recent advancements in experimental techniques, such as atomic force microscopy (AFM) and surface forces apparatus (SFA), have significantly improved our ability to study and quantify molecular and colloidal forces under different media and temperature conditions (Tabor et al. 2014; Toca‐Herrera 2019). The first direct measurement of hydrophobic interactions was reported as a long‐range force, stronger than the van der Waals (vdW) dispersion, that decays exponentially with distance (Israelachvili and Pashley 1982). Following this work, after decades of research (Pashley et al. 1985; Rabinovich and Yoon 1994; Yoon et al. 1997; Craig et al. 1999; Nguyen et al. 2003; Wang and Yoon 2008; Donaldson et al. 2011; Donaldson Jr. et al. 2013; Tabor et al. 2013; Soga et al. 2015; Stock et al. 2015), a generic interaction potential was proposed based on empirical results, derived when studying forces between stressed surfactant/lipid bilayers (Donaldson Jr. et al. 2015). This generic potential is expressed as an exponential decay function with decay lengths ranging from 0.3 to 1 nm, and a pre‐exponential factor relating the interfacial tension between water and the hydrophobic phase, as well as the fraction of the hydrophobic area.

However, the physical origin of the hydrophobic interaction has remained a subject of ongoing investigation (Zeng et al. 2015; Wang et al. 2023). The variety of settings, equipment limitations, control of conditions, complexity of system properties, difficulty of preparing reproducible surfaces, and correct quantification of interactions has often resulted in conflicting experimental results. Proposed mechanisms focus on the behavior of water molecules between non‐polar surfaces at short separations (*D* < 10 nm), namely polarization and entropic effects attributed to orientational correlations, proton‐hopping, and hydrogen‐bonding, amongst others (Despa and Berry 2007; Hammer et al. 2010; Kanth et al. 2010; Shelton 2012; Hassanali et al. 2013). Interestingly, force‐distance measurements between hydrophobic surfaces in H‐bonding solvents (e.g., ethanol) disclosed the presence of a solvophobic interaction (Wang et al. 2012; Li and Yoon 2014). This interaction has been attributed to entropy decreasing due to H‐bonding structure formation. Nevertheless, the extensive length of some of the observed interaction ranges (> 100 nm) might be indicative of bubble coalescence, widely reported in the literature (Ishida, Inoue, et al. 2000; Ishida, Sakamoto, et al. 2000; Zhang et al. 2006; Hampton et al. 2008; Lohse and Zhang 2015), also observed in non‐H‐bonding solvents (Considine and Drummond 2000). Micro‐ and nanobubble bridging has been distinguished from intrinsic hydrophobic interaction, being dependent on gas solubility and surface roughness (Nalaskowski et al. 1999; Zhang et al. 2004; Azadi et al. 2015). Moreover, polarization effects suggest that the intrinsic hydrophobic interaction might have a similar nature to van der Waals forces. As the polarity of the media is a parameter of vdW forces, it is of interest to study its influence in a hydrophobic system. This way, varying progressively media polarity with aqueous‐solvent binary mixtures is a feasible strategy. Nonetheless, probing experiments between symmetric hydrophobic surfaces in different water‐solvent binary mixtures are scarce, mainly performed in water‐ethanol (Nguyen et al. 2003; Wang et al. 2012; Hansson et al. 2013; Soga et al. 2015; Schrader et al. 2015).

In this primer, we present a straightforward method to study molecular interactions between hydrophobic surfaces in water and water:DMSO mixtures. We report new findings concerning how lowering the polarity of the solvent affects the interaction between fluorocarbon surfaces. Our objective is to provide a simple protocol for the quantification of the hydrophobic interaction. Thus, we explain how to functionalize the substrates, use a suitable probe and establish the correct settings for force‐distance measurements with AFM. Additionally, we describe the steps for processing and analyzing AFM data and fitting with an extended van der Waals model.

## 2 Materials and Methods

### 2.1 AFM Measurements

Atomic force microscopy (AFM) force‐distance measurements were performed with both a Nanowizard 1 AFM (Bruker, Germany) and a Nanowizard 3 AFM (Bruker, Germany) using contact mode at room temperature (ca. 295 K). The cantilevers used were triangular silicon nitride with a pyramidal tip of 10 nm nominal radius (DNP‐S10, A, Bruker) and gold‐coated (50 nm Au) rectangular with a pyramidal tip of 20 nm nominal radius (PR‐UM‐TNIR‐D‐10, Bruker). The thermal noise method was used to estimate the spring constant of the cantilevers (Hutter and Bechhoefer 1993). The measured thermal noise spectra can be found in Figure S1. Triangular cantilevers exhibited a mean spring constant of 0.37 ± 0.01 N m^−1^ whereas rectangular cantilevers exhibited a mean spring constant of 12.3 ± 0.8 N m^−1^. At least 100 force‐distance curves were conducted in a 10 μm × 10 μm grid with a 1 μm spacing between measurement positions. The probing speed was fixed to 100 nm s^−1^. For studying the main systems, measurements were carried out at least thrice in an open stage by depositing a drop of solvent on the sample and cantilever. Each measurement replica was conducted with a different batch of prepared systems. The media used were Milli‐Q water, dimethyl sulfoxide 99.5% (DMSO, Honeywell Riedel‐de‐Haën), and water:DMSO laboratory‐prepared solutions of 3:1, 2:1, and 1:1 M ratios.

### 2.2 AFM Cantilever Functionalization

Silicon nitride triangular and gold rectangular cantilevers were cleaned in UV/ozone for 1 h prior to functionalization. Cleaned silicon nitride triangular cantilevers were coated with 3 nm of chromium and then with a top layer of 10 nm of gold using a sputter coater. Both gold rectangular and gold‐coated triangular cantilevers were then placed in a desiccator chamber next to an open vessel filled with 3 mL of 1H,1H,2H,2H‐Perfluorooctanethiol 97% (FOTT, AB229378, abcr GmbH, Germany). The pressure in the chamber was reduced using a Laboport N96 pump (KNF, Germany) for a few minutes and the chamber was then kept sealed at room temperature (ca. 295 K) for 19 h. Gold‐coated triangular cantilevers and rectangular cantilevers were cleaned with UV/ozone for 1 h and used as control probes.

### 2.3 Substrate Functionalization

Coverslips were rinsed with ethanol and dried with N_2_. The coverslips were then oxygen plasma cleaned for 10 min. The cleaned coverslips were then coated with 3 nm of chromium and then with a top layer of 10 nm of gold using a sputter coater. Next, the gold‐coated coverslips were placed in a desiccator chamber beside an open vessel filled with 3 mL of 1H,1H,2H,2H‐Perfluorooctanethiol 97% (FOTT, AB229378, abcr GmbH, Germany). The pressure in the chamber was reduced for a few minutes using a Laboport N96 pump (KNF group, Germany) and then sealed at room temperature (ca. 295 K) for 19 h. Afterwards, the samples were rinsed with ethanol and dried with N_2_. Gold‐coated coverslips were UV/ozone cleaned for 1 h and used as control substrates.

### 2.4 Sample Characterization by Contact Angle

FOTT functionalized substrates were characterized using the drop sessile method. Measurements were performed at least three times with 10 μL Milli‐Q water drops at room temperature (ca. 295 K). The drop was deposited on the sample using a micropipette. The same measurements were carried out on EtOH‐cleaned and N_2_‐dried gold‐coated substrates and in UV/ozone‐cleaned gold‐coated substrates as controls. Contact angles were determined using a Kruess EasyDrop instrument (Kruess, Hamburg, Germany).

### 2.5 AFM Data Processing, Averaging and Error Estimation

The determination of the contact point (i.e., distance zero) and correction of the piezo scanner height was performed with JPK Data Processing SPM‐5.0.133 software (JPK Instruments AG, Germany). We identified the contact point at the beginning of the linear regime of the piezo height and cantilever vertical deflection for both FOTT‐modified and bare substrates, assuming them as infinitely hard materials.

Every system set of N‐force‐distance curves (ca. 10,000 points per curve) was averaged to a 1000 points curve using the linear interpolation method and common‐x range algorithm of OriginPro software (OriginLab Corporation, USA). Representative averaged curves and raw data can be consulted in Figure S2. Afterwards, the mean curve was averaged with its respective averaged replicas using the same methodology. The standard error was also calculated. The resulting mean curve was used for model fitting. The error estimation of fitted parameters (e.g., Hamaker constant) was carried out with the Lavenberg–Marquardt algorithm.

### 2.6 Force–Distance Curve Simulation, Data Fitting and Constant Calculation

Van der Waals (vdW) dispersion forces are present in all atoms and molecules (Israelachvili 2011a, 2011b) and therefore are crucial for the interpretation and modeling of measured force‐distance curves with atomic force microscopy. In this manner, straightforward expressions have been derived for fitting AFM experimental data. Thus, Equation (1) describes nonretarded vdW forces at short separations between a pyramidal cantilever with a tip and a flat surface across a medium (Zanette et al. 2000)

(1) $$F=-\frac{A_{H}R}{6D^{2}}$$

The vdW forces are always attractive between identical materials across a medium (Israelachvili 2011a, 2011b), where *A*_*H*_ is the nonretarded Hamaker constant, *R* is the radius of the tip and *D* is the tip‐sample separation. Lifshitz theory (Lifshitz 1956) allowed the calculation of Hamaker constants of bodies interacting in a medium. The theory solves the additivity problem, treating molecules as a continuum that interacts based on bulk properties (i.e., dielectric constant and refractive index). Therefore, Hamaker constants were calculated for a system of two identical dielectric materials (1) interacting across a medium (3), described in Equation (2) (Israelachvili 2011a, 2011b)

(2) $$A_{H}=\frac{3}{4}k_{B}T{\left(\frac{\varepsilon_{1}-\varepsilon_{3}}{\varepsilon_{1}+\varepsilon_{3}}\right)}^{2}+\frac{3h\nu_{e}}{16\sqrt{2}}\frac{{\left(n_{1}^{2}-n_{3}^{2}\right)}^{2}}{{\left(n_{1}^{2}+n_{3}^{2}\right)}^{3/2}}$$

here, the first term corresponds to the dipolar contributions and the second to the dispersion contribution, where *k*_*B*_ is the Boltzmann constant, *T* is the temperature, *ε* is the dielectric constant, n is the refractive index, *h* is the Planck's constant, and *ν*_*e*_ is the electronic absorption frequency in the UV region, normally 3 × 10^15^ s^−1^, which corresponds to the ionization of molecules (Butt et al. 2005; Israelachvili 2011a, 2011b). Thus, the Hamaker constants were calculated for a symmetric system of fluorocarbon (1) interacting across water (3) and DMSO (4) at 298.15 K using the physical constants of Table 1, obtaining *A*_*H*131_ = 6.7 × 10^−21^ J and *A*_*H*141_ = 2.7 × 10^−21^ J, respectively. Although a practical expression, note that Equation (2) assumes that the absorption frequencies of all the media are the same.

**TABLE 1:** Parameters used to calculate Hamaker constants using Equation (2).

Medium	Dielectric constant, *ε*	Refractive index, *n*	References
Water	78.4	1.33	Kaatze et al. (1989); LeBel and Goring (1962)
DMSO	47.0	1.47	Kaatze et al. (1989); LeBel and Goring (1962)
Fluorocarbon	1.90	1.45	Takenaga et al. (2008)

Nevertheless, Equation (2) cannot be used for metals interacting across a medium, as the dielectric constant of a conductor tends to infinity (Butt et al. 2005). For instance, the Hamaker constant for the metal–metal interaction in vacuum has been calculated using the plasma frequencies of metals, *A*_*H*_≈40 × 10^−20^ J (Israelachvili 2011a, 2011b). Furthermore, estimations of the gold–gold interaction across water have been calculated from optical reflectance data (Parsegian and Weiss 1981). The authors fitted the data to the frequency‑dependent linear dielectric response of the material’s electric field, assuming a *ε*_vis_ = *n* ^2^_vis_ constraint. They obtained Hamaker values in the (90–300) × 10^−20^ J range, which might not be interpreted physically as per their formal mathematical approach. Discordantly, experimental data disclosed much lower Hamaker constant values, (7–25) × 10^−20^ J (Biggs and Mulvaney 1994; Larson et al. 1997; Kane and Mulvaney 1998). The disparity between experimental and calculated *A*_*H*_ could originate from theoretical oversimplifications and frequency restricted data (Tolias 2018). In this regard, the use of extended dielectric data and the Kramers–Kronig relation has provided calculated values closer to empirical values, *A*_*H*_ (gold–water–gold) = 15.7 × 10^−20^ J (Jiang and Pinchuk 2016).

Despite their unknown origin, hydrophobic interactions (H) are longer‐ranged and stronger than vdW forces. For short separations between hydrophobic surfaces (*D* < 10 nm), proposed mechanisms feature water related phenomena (e.g., polarization and entropic effects) at the interface and bulk (Despa and Berry 2007; Hammer et al. 2010; Kanth et al. 2010; Shelton 2012; Hassanali et al. 2013). Reported direct force‐distance measurements between hydrophobic surfaces in water exhibit an exponential decay, generally described with Equation (3) (Israelachvili and Pashley 1982; Donaldson Jr. et al. 2015)

(3) $$F=-R\;C\;\exp^{-\frac{D}{\lambda }}$$

where *C* and *λ* are a pre‐exponential constant and a decay length, respectively, both dependent of media properties. The combination of Equations (1) and (3) allowed the fitting of our experimental data. The calculations were performed using OriginPro 2024 software (Originlab corporation, USA).

### 2.7 System Preparation and Characterization

To prepare hydrophobic surfaces we used a fluorocarbon reagent (FOTT, 1H,1H,2H,2H‐Perfluorooctanethiol). In fact, FOTT is the thiol molecule analog to the silane FOTS, which we used in our previous work to functionalize silica particles and study its colloidal interactions (Ponce‐Gonzalez et al. 2025). Available nanofabricated gold cantilever probes allowed us to functionalize molecular systems (i.e., tips with nanometric radius).

It is known that thiol molecules form stable self‐assembled monolayers (SAM) on gold at ambient conditions (Vericat et al. 2010). Despite the high affinity between sulfur atoms and metal surfaces, the adsorption mechanism (i.e., physisorption or chemisorption) is still a debate (Xue et al. 2014; Inkpen et al. 2019). Hydrocarbon SAMs have been prepared in several AFM studies, employing gold dip‐coating methods (Kane and Mulvaney 1998; Wang and Yoon 2008; Li and Yoon 2014; Stock et al. 2015). However, this procedure requires careful control of reaction time, thiol concentration, and solvent, as well as a thorough workup to remove unbound thiols, which can lead to contamination, low reproducibility, and AFM measurement artifacts. Therefore, as FOTT is relatively volatile, we employed chemical vapor deposition (CVD) at room temperature (ca. 295 K) and reduced pressure to establish a simple technique for obtaining molecularly smooth surfaces for AFM studies without the aforementioned drawbacks.

FOTT deposition time studies on gold (Figure S3A) showed that after 19 h, the water contact angle disclosed a value of 96° ± 3° (Figure S3B). For instance, contact angles greater than 90° are considered hydrophobic (Law 2014). Note that we performed the FOTT‐CVD on ozone/UV‐cleaned gold surfaces, which exhibited a contact angle close to zero degrees (Figure S3C). In this regard, it has been found that the high hydrophilicity of cleaned gold is due to the surface formation of an oxide thin film (King 1995; Hattori and Kitamura 2023). Comparatively, control gold samples cleaned just with ethanol (Figure S3D) showed much higher contact angles (61° ± 3°) due to ambient contamination (e.g., adventitious carbon). In this matter, the hydrophilic nature of gold is still an open discussion (Smith 1980).

### 2.8 Detection of Forces at Nanometric Range

The quantification of van der Waals (vdW) forces and hydrophobic interactions, in symmetric surfaces across a solvent, requires adequate detection conditions. These phenomena have such a short‐ranged strong attractive nature that they cannot be measured with soft AFM cantilevers. In our last primer, we observed a cantilever jump‐in when probing a hydrophobic surface in water at a separation distance of about 3–4 nm (Ponce‐Gonzalez et al. 2025). When the gradient of the attractive force overcomes the spring constant (k) of the cantilever (dF/dz > k), the probe is no longer stable and leaps in to the surface. Consequently, no data can be obtained from the force‐distance measurement within this range, precluding the discrimination between vdW forces and hydrophobic interaction, so subsequent quantification. For instance, the jump‐in phenomenon has been a recurrent problem in a myriad of AFM studies (Wu 2010; Rodrigues et al. 2012). Nevertheless, some works have partially tackled this issue, obtaining exponentially decaying data before the jump‐in (Stock et al. 2015; Xie et al. 2016).

**FIGURE 1:**
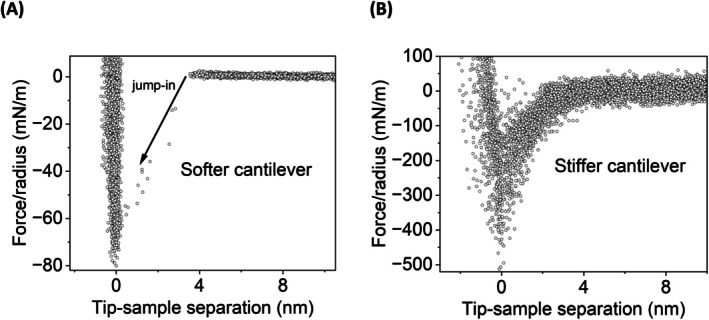
Force‐distance curves (*N* = 80, approaching segment) measured in the symmetric system Au‐FOTT in water with a softer cantilever (A) and a stiffer cantilever (B) with an average spring constant of 0.37 ± 0.01 N m^−1^ and 12.3 ± 0.8 N m^−1^, respectively. The experiments were performed with a probing speed of 100 nm s^−1^ and at room temperature (ca. 295 K).

In the actual work, we followed two strategies to decrease these strong attractive forces and interactions. First, instead of using a hydrophobic colloidal probe with a micrometric radius (Ponce‐Gonzalez et al. 2025), we used a tip with a nanometric radius. However, Figure 1A shows how the jump‐in at ca. 4 nm persists when reducing the radius of interaction (i.e., size of the tip). On the other hand, using a stiffer cantilever with a tip of a similar radius conferred enough stability to the probe for measuring the attractive forces at the short range without snapping into contact (Figure 1B).

## 3 Results and Discussion

### 3.1 Attractive Interactions in Water

To study van der Waals (vdW) forces and hydrophobic interactions, we conducted AFM force‐distance experiments on a symmetrical system comprising a gold tip probe with a nanometric radius (*R* = 20 nm) and a gold‐coated coverslip, both functionalized with a fluorocarbon SAM (i.e., FOTT). The measurements were performed in water with a probing speed of 100 nm s^−1^ at room temperature (ca. 295 K). Analogous experiments were carried out on a bare gold symmetric system cleaned with ozone/UV.

**FIGURE 2:**
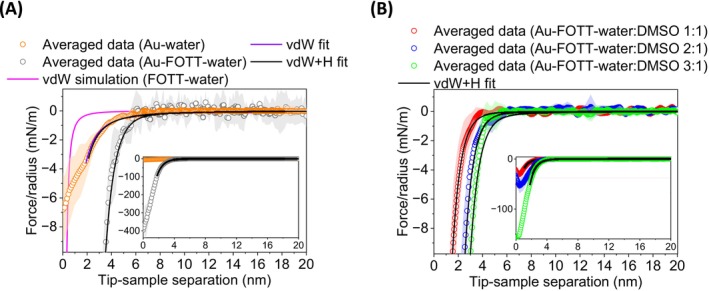
Radius normalized average force–distance curves (approaching segment) between gold tip and gold sample (hollow orange dot) and FOTT‐coated gold tip and FOTT‐coated gold sample (gray hollow dot) across water (A), and (B) across water:DMSO 3:1 (hollow green dot), water:DMSO 2:1 (hollow blue dot) and water:DMSO 1:1 (hollow red dot) molar ratio. Purple, black and pink lines represent vdW fit, vdW+H fit and vdW simulation, respectively. The color‐shaded areas correspond to the standard error of each averaged curve. The inset plots represent a *y*‐axis widening of the same data. Note that although gold and FOTT‐coated gold f–d curves are set to the same *d* = 0, the contact point for the FOTT‐coated gold substrate has to be accounted by two times the thickness of the adsorbed fluorocarbon layer. The experiments were performed with a probing speed of 100 nm s^−1^ and at room temperature (ca. 295 K).

As shown in Figure 2A, the attractive interaction observed in the FOTT‐water‐FOTT system is stronger and longer‐ranged than the simulated vdW force with the corresponding calculated Hamaker constant for a fluorocarbon‐water‐fluorocarbon system (*A*_*H*_ = 6.7 × 10^−21^ J). Thus, we identified the experimental interaction as the hydrophobic attraction. Hereby, we can exclude other possible contributions (e.g., electrostatics, surface roughness). In this matter, for electrostatics, we should expect a repulsive interaction, which was not observed in the force‐distance curves. Further, it is worth mentioning that hydrophobic surfaces with micrometric surface roughness (e.g., polystyrene) have a contribution and are longer‐ranged (e.g., 35–85 nm) due to nucleation and coalescence of bubbles (Nalaskowski et al. 1999; Zhang et al. 2006; Ponce‐Gonzalez et al. 2025).

Then, we fitted the data with an extended vdW model, which includes the hydrophobic interaction term (H), by means of a combination of Equations (1) and (3). Table 2 shows the returned parameters from the fittings. For instance, the FOTT–FOTT system exhibited a hydrophobic constant of *C* = 1.02 N m^−1^, a much higher value in comparison to the interfacial tension (*γ*) of a liquid fluorocarbon in water, 54.6 mN m^−1^ (Morgado et al. 2020). However, if we consider *C* = 4π*γH*_*y*_ as from Donaldson's general potential (see Section 4 of Supporting Information), where *H*_*y*_ = 1 is the Hydra parameter for a fully hydrophobic surface (Donaldson Jr. et al. 2015), we obtain a similar magnitude value to the experimental one, *C* = 0.69 N m^−1^. The returned decay length was *λ* = 0.74 nm, which is within the range of other quantified hydrophobic interactions (Donaldson Jr. et al. 2015; Stock et al. 2015; Xie et al. 2016). Accordingly, the gold–gold system disclosed a much lower hydrophobic constant (i.e., *C* < 0.01 N m^−1^) as per its hydrophilic nature after the ozone/UV cleaning treatment. Interestingly, the Hamaker constant for both symmetric systems of bare gold and hydrophobized gold across water fitted the same value (i.e., *A*_*H*_ = 7.50 × 10^−20^ J), as if the bulk material (gold) dominated the van der Waals forces independently of the adsorbed FOTT monolayer. For instance, fitting of the gold‐water‐gold data with the vdW model (Equation 1) returned a Hamaker constant of (7.80 ± 0.10) × 10^−20^ J (*R* ^2^ = 0.97), a value close to the calculated with spectral data (Jiang and Pinchuk 2016).

**TABLE 2:** Values of the obtained parameters with the fittings of the van der Waals (vdW) extended model.

Symmetrical system	Hamaker constant, *A*_*H*_ (×10^−20^ J)	Hydrophobic constant, *C* (N m^−1^)	Decay length, *λ* (nm)	Regression coefficient, *R* ^2^
Au‐water	7.50 ± 0.34	< 0.01	0.82 ± 0.21	0.97
Au‐FOTT‐water	7.50 ± 1.24	1.02 ± 0.01	0.74 ± 0.01	0.99
Au‐FOTT‐water:dmso (3:1 M ratio)	5.00 ± 0.66	0.66 ± 0.01	0.70 ± 0.01	0.99
Au‐FOTT‐water:dmso (2:1 M ratio)	2.00 ± 0.57	0.49 ± 0.02	0.65 ± 0.01	0.99
Au‐FOTT‐water:dmso (1:1 M ratio)	1.00 ± 0.19	0.10 ± 0.01	0.67 ± 0.01	0.99

As commented previously (Section 2.6), fluorocarbon‐fluorocarbon and metal–metal Hamaker constants across water differ approximately by one order of magnitude. For this matter, models have been derived for predicting the vdW force of a multi‐layered system (Section S5). Furthermore, the presence of retarded vdW forces was also evaluated (Section S6) and confirmed for the gold–gold system in water (Figure S5). Moreover, note in Figure 2A that after exponentially decaying attractive forces, an attractive linear regime was observed at very close separations (ca. 2 nm). Correspondingly, this strong short‐range attractive force has been attributed to water structuring effects at the water‐hydrophobic interface (Hammer et al. 2010).

### 3.2 DMSO Influence in the Interactions

After identifying and quantifying the hydrophobic interaction (H) between the surfaces of a fluorocarbon‐fluorocarbon system across water (FOTT–water–FOTT), we performed force‐distance experiments under the same conditions but in different binary solvent mixtures of water:DMSO molar ratios. This allowed us to study the overall influence of a progressive change in polarity in one of the three interacting media.

Figure 2B shows how the different DMSO molar ratios in the system barely affect the decay length of the exponential region of the attractive interactions, varying 0.65–0.70 nm (Table 2). The physical meaning of the decay length has been related to molecular size in oscillatory force models (Evans and Wennerström 1999). Nevertheless, the distance at which the decay starts is more pronounced, following a decreasing trend when increasing DMSO concentration (see Figure S6 with derived f–d curves). Accordingly, hydrophobic (*C*) and Hamaker (*A*_*H*_) constants decreased when DMSO concentration increased, as if they were cross‐talking, as the strength of the attractive interaction was lowering (see Table 2). This decreasing of the Hamaker constant agrees with the calculated constants for a fluorocarbon‐water‐fluorocarbon and a fluorocarbon‐DMSO‐fluorocarbon (see Section 2.6). Consistently, authors reported the decreasing of the Hamaker constant for a lipid‐water‐lipid system when increasing mol% DMSO (Schrader et al. 2015). This way, decreasing the medium polarity where two identical bodies interact decreases the Hamaker constant. For instance, experimental evidence was also reported for gold‐water‐gold and gold‐ethanol‐gold systems (Kane and Mulvaney 1998).

On the other hand, the decrease of the pre‐exponential hydrophobic constant C with increasing DMSO concentration can be explained by the decrease of the surface‐liquid interfacial tension. The physical meaning of *C* (see Section 4 of the Supporting Information) has been related to the interfacial tension between the hydrophobic phase and water (Donaldson Jr. et al. 2015). For instance, adhesion and contact angle studies on hydrophobic surfaces report a decrease in solid–liquid interfacial tension from water to DMSO (Vezenov et al. 2002; Mondal et al. 2015; Zhang et al. 2019).

It is worth remarking that our measurements were consecutively performed for every replica from less to more DMSO concentration. As a SAM stability control, after measuring a FOTT–FOTT system consecutively in water, water:DMSO 3:1, 2:1, 1:1 and DMSO (Figure S7A), it was measured in water again, increasing back the strength of the attractive interaction (Figure S7B). In this sense, the fluorocarbon SAM on gold should be stable under these conditions, as DMSO prevents the desorption of thiols (Yang et al. 2004). Nonetheless, we were not able to reproduce the FOTT–FOTT attractive interactions in DMSO in different replicas (Figure S8). For this reason, the FOTT–FOTT system was also investigated in DMSO with a softer cantilever exhibiting more reproducible results in detecting attractive interactions (Figure S9A). On that account, a softer cantilever might have more sensitivity in a higher viscous solvent such as DMSO. Intriguingly, we detected attractive interactions stronger than vdW forces (Figure S9B). When this system was measured in water (see Section 2.8, Figure 1A), a jump‐in was observed. Regarding this, we believe the recorded attractive forces in DMSO might be due to short‐range solvophobic interaction. Additionally, our control experiments on a gold‐DMSO‐gold system exhibited repulsive interactions (Figure S10).

Also noting that the attractive linear regime observed at very close separations in FOTT‐water‐FOTT (ca. 2 nm) was also in the presence of DMSO. Following, this steep regime reached a minimum, difficult to differentiate in some systems from the contact point. The surpass of the minimum well before the contact point could be caused by adhesion and indentation, as the tip and sample can deform upon contact (Butt et al. 2005).

## 4 Conclusions

In this primer, we have presented how to quantify the influence of water‐solvent binary mixtures on molecular interactions between hydrophobic surfaces using atomic force microscopy (AFM). Hydrophobic surfaces were easily prepared by the chemical vapor deposition of a fluorocarbon thiol (i.e., FOTT) on gold, obtaining a self‐assembled monolayer (SAM). The use of a stiff cantilever (ca. 12 N m^−1^) allowed the quantification of van der Waals (vdW) forces and hydrophobic interactions (*H*).

Force‐distance measurements in water between FOTT‐coated gold surfaces and bare gold surfaces disclosed that the hydrophobic interaction is longer‐ranged and stronger than theoretical van der Waals forces. Thus, the data was fitted to an extended vdW model, including the *H* contribution, to calculate parameters such as the Hamaker constant and the hydrophobic decay length. Experiments in water:DMSO mixtures showed how both vdW attractive forces and attractive hydrophobic interactions decrease as the polarity of the medium decreases. Furthermore, assays in pure DMSO between FOTT surfaces disclosed the presence of a solvophobic attractive interaction.

Future studies include new experiments to be performed in organic solvents with different polarity and dispersive properties, as well as H‐bonding and non‐bonding nature. Additional studies will target modulation of hydrophobic surface polarity rather than solvent composition, using approaches such as applied electric potentials or light irradiation.

## Author Contributions

**Luis N. Ponce‐Gonzalez:** conceptualization, investigation, writing – original draft, methodology, validation, formal analysis, data curation, visualization, software. **José L. Toca‐Herrera:** conceptualization, funding acquisition, writing – review and editing, validation, formal analysis, project administration, supervision, resources, methodology.

## Funding

This work was supported by the Austrian Science Fund (P35777‐B).

## Supporting information


**Data S1:** Supporting Information.

## Data Availability

The data that support the findings of this study are available from the corresponding author upon reasonable request.
