# Supplementary material for: Measuring Molecular Forces With Atomic Force Microscopy 1: Solvent Influence on Hydrophobic Interactions
Source: Microsc Res Tech. 2025 Dec 19;89(5):718–26. doi: 10.1002/jemt.70111 (PMC13048189; doi:10.1002/jemt.70111)
Supplement: Supplementary file 1 — Data S1: Supporting Information. [file JEMT-89-718-s001.docx]

**Supporting Information**

**Measuring molecular forces with atomic force microscopy 1: solvent influence on hydrophobic interactions**

Luis N. Ponce-Gonzalez,^1^ and José L. Toca-Herrera*^1^

^1^BOKU University, Institute of Biophysics, Muthgasse 11, 1190 Vienna, Austria.

Email: [jose.toca-herrera@boku.ac.at](mailto:jose.toca-herrera@boku.ac.at)

Contents

[Section 1: thermal noise spectra 1](#_Toc211270099)

[Section 2: raw force-distance curves with average 2](#_Toc211270100)

[Section 3: contact angle measurements 3](#_Toc211270101)

[Section 4: Donaldson general potential for hydrophobic interactions 4](#_Toc211270102)

[Section 5: force-distance curve simulation with multi-layer van der Waals model 5](#_Toc211270103)

[Section 6: van der Waals retardation model fitting 6](#_Toc211270104)

[Section 7: force-distance curves and their first derivatives 7](#_Toc211270105)

[Section 8: control measurements 8](#_Toc211270106)

[References 10](#_Toc211270107)

# Section 1: thermal noise spectra

| **(A)**   | **(B)**   |
| --- | --- |

**Figure S1.** Representative thermal noise spectra disclosing the resonance frequency peak of the soft (A) and stiff (B) cantilevers used for the force-distance measurements. The measurements were carried out in water at room temperature (ca. 295 K).

# Section 2: raw force-distance curves with average

| **(A)**  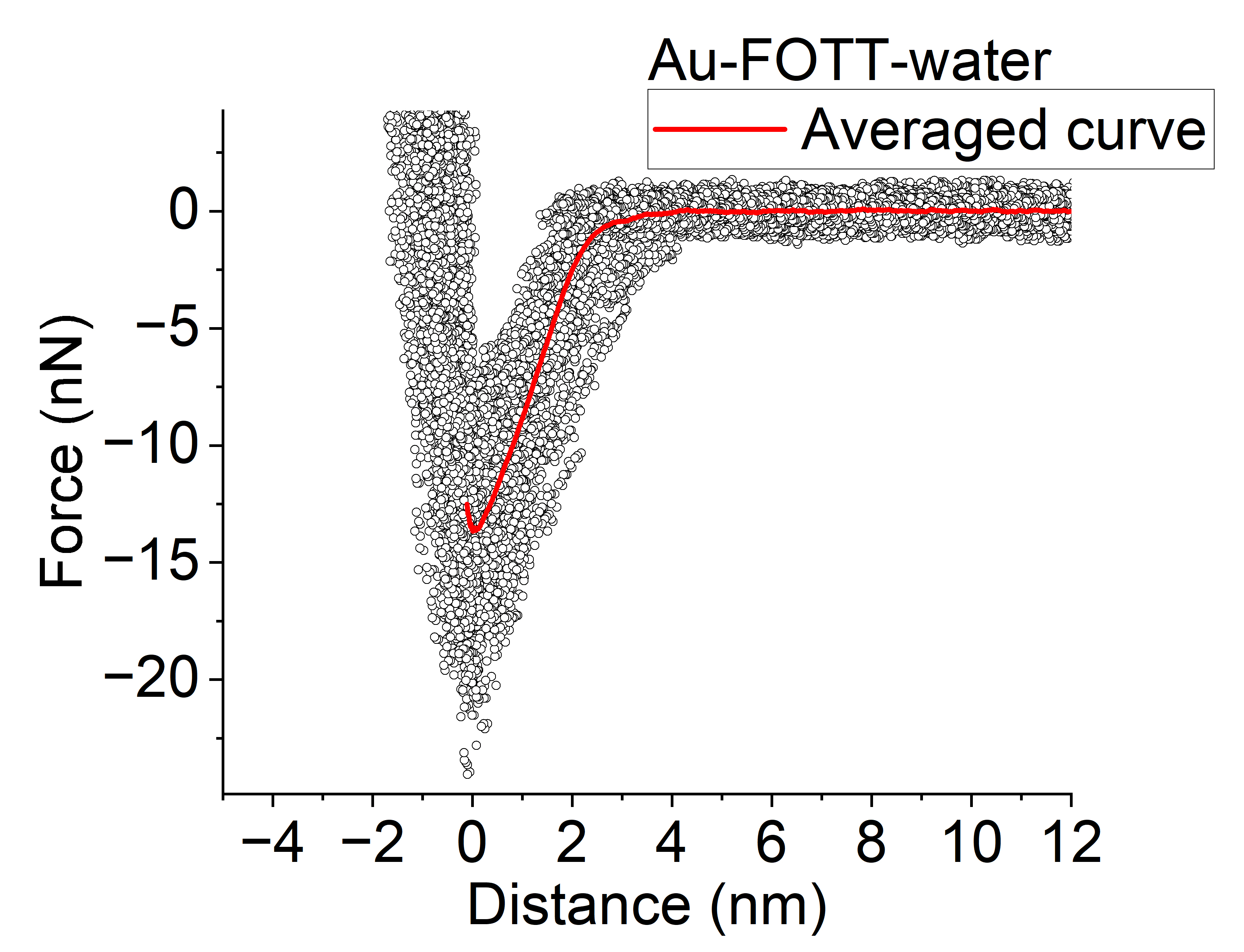 | **(B)**  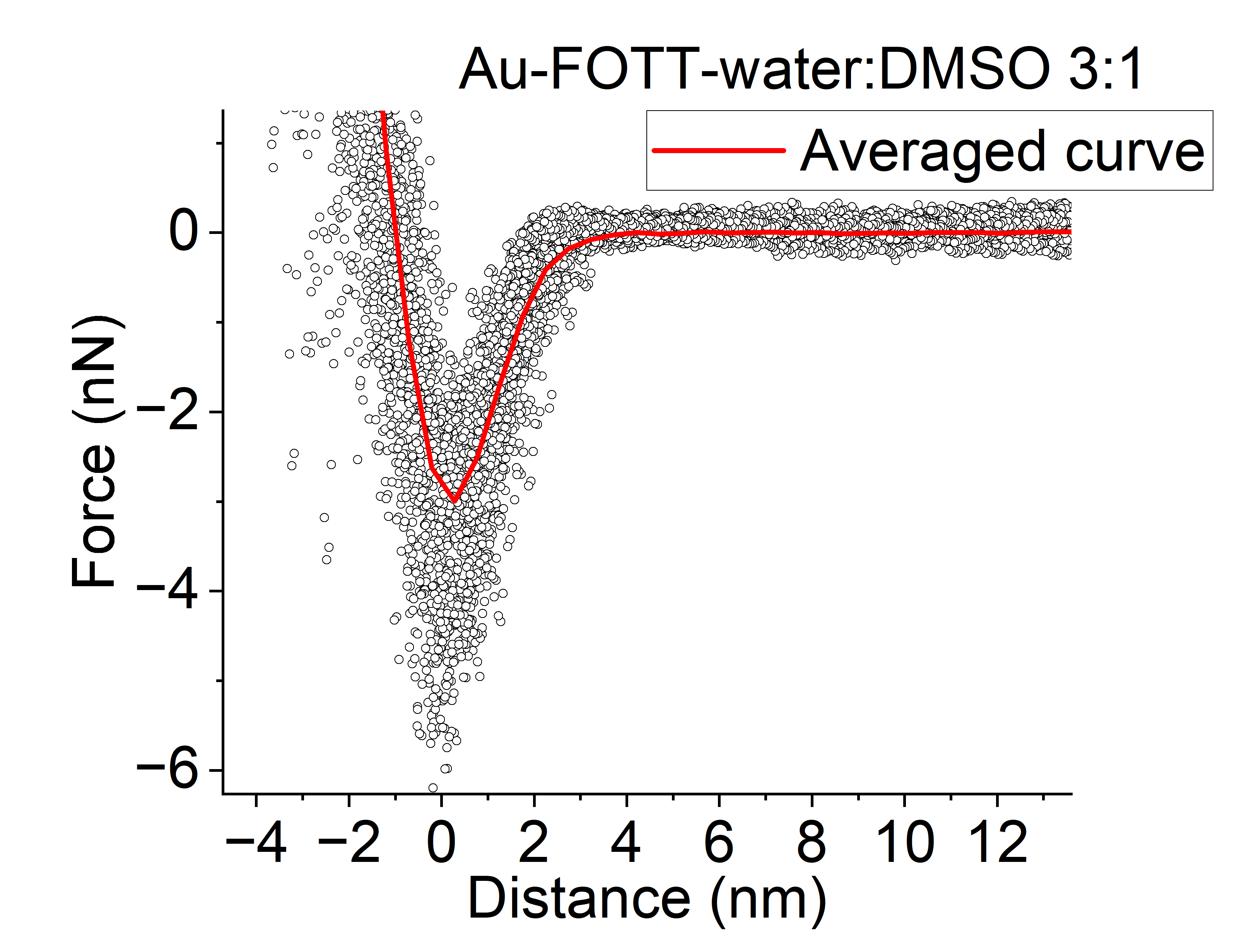 |
| --- | --- |
| **(C)**  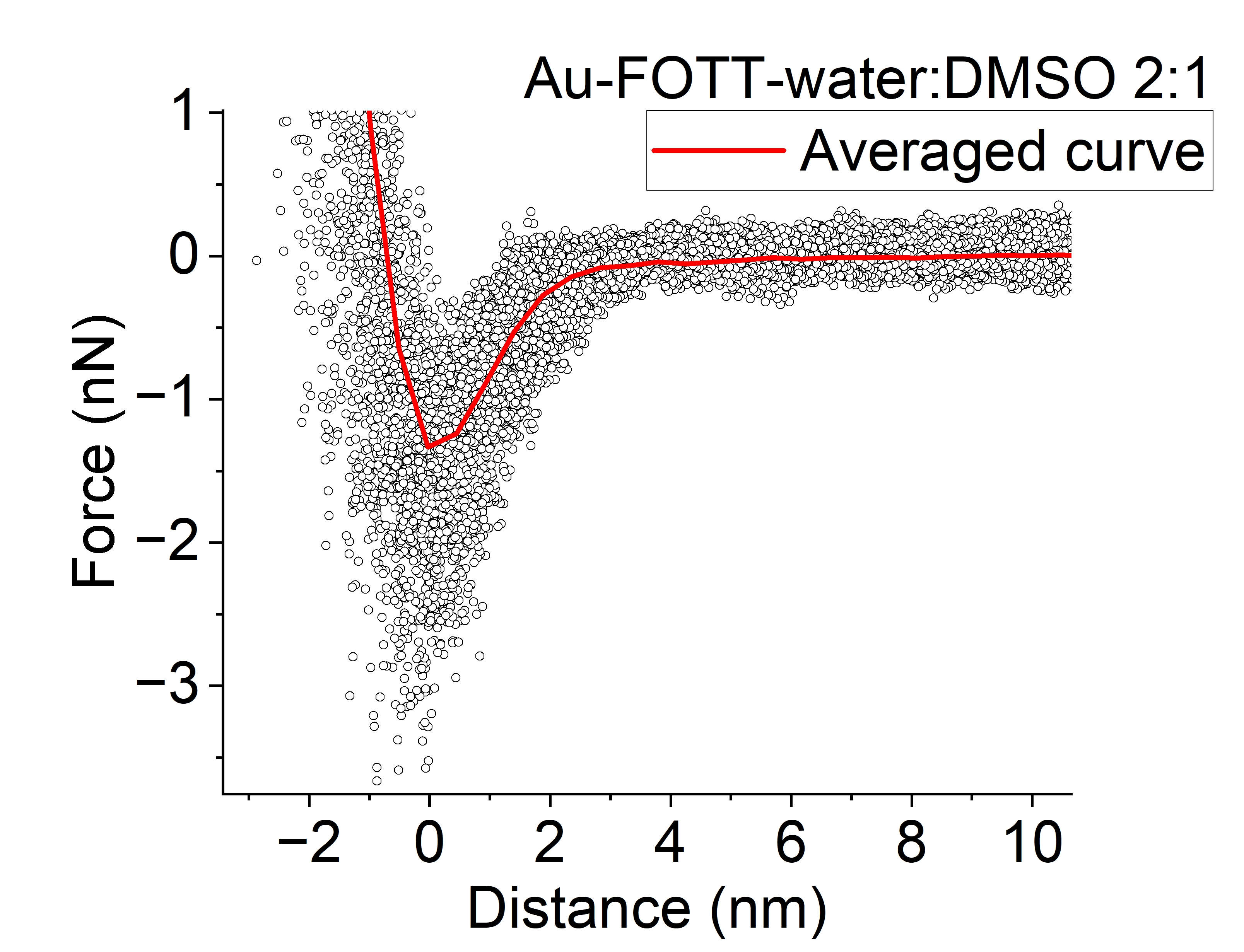 | **(D)**  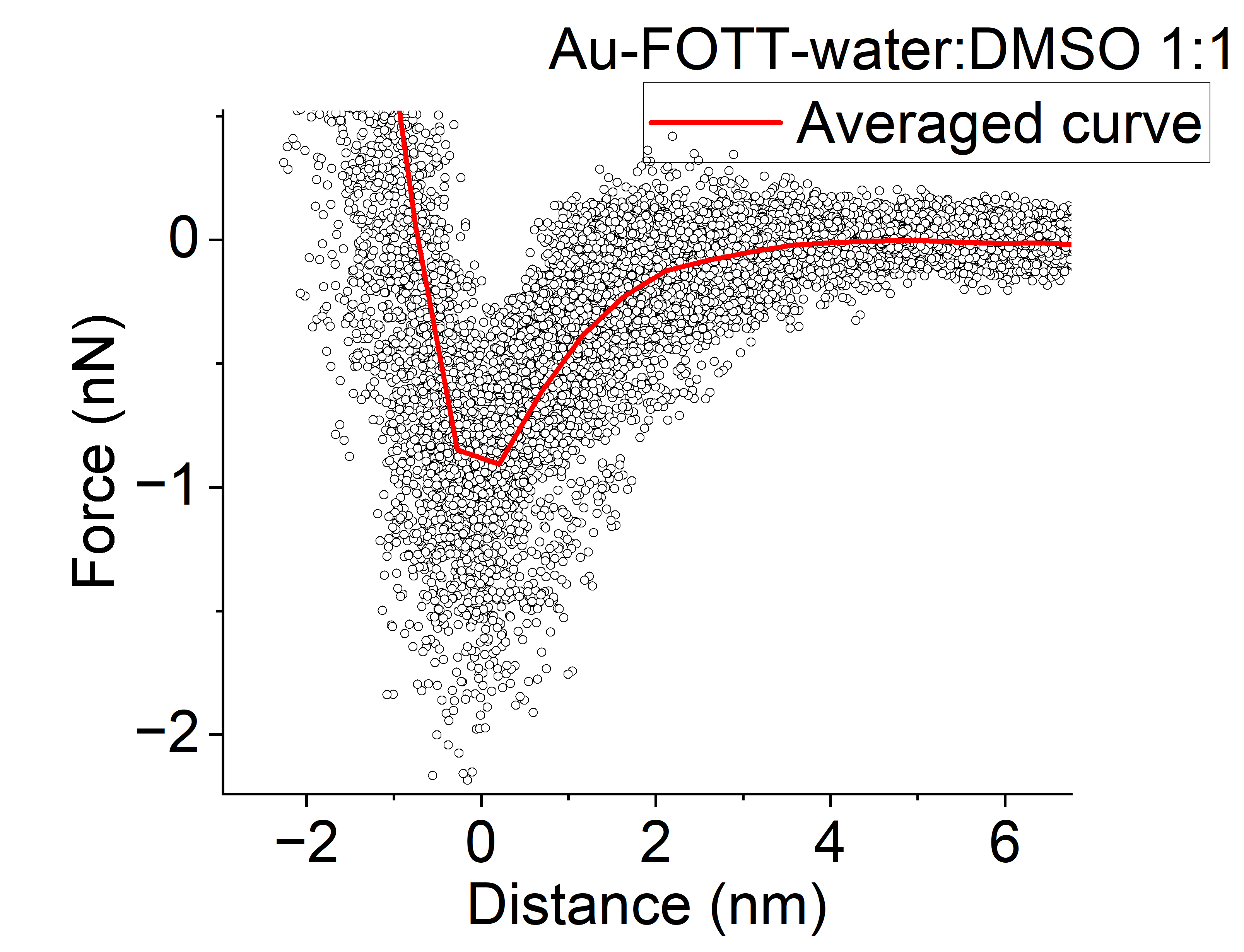 |
| **(E)**  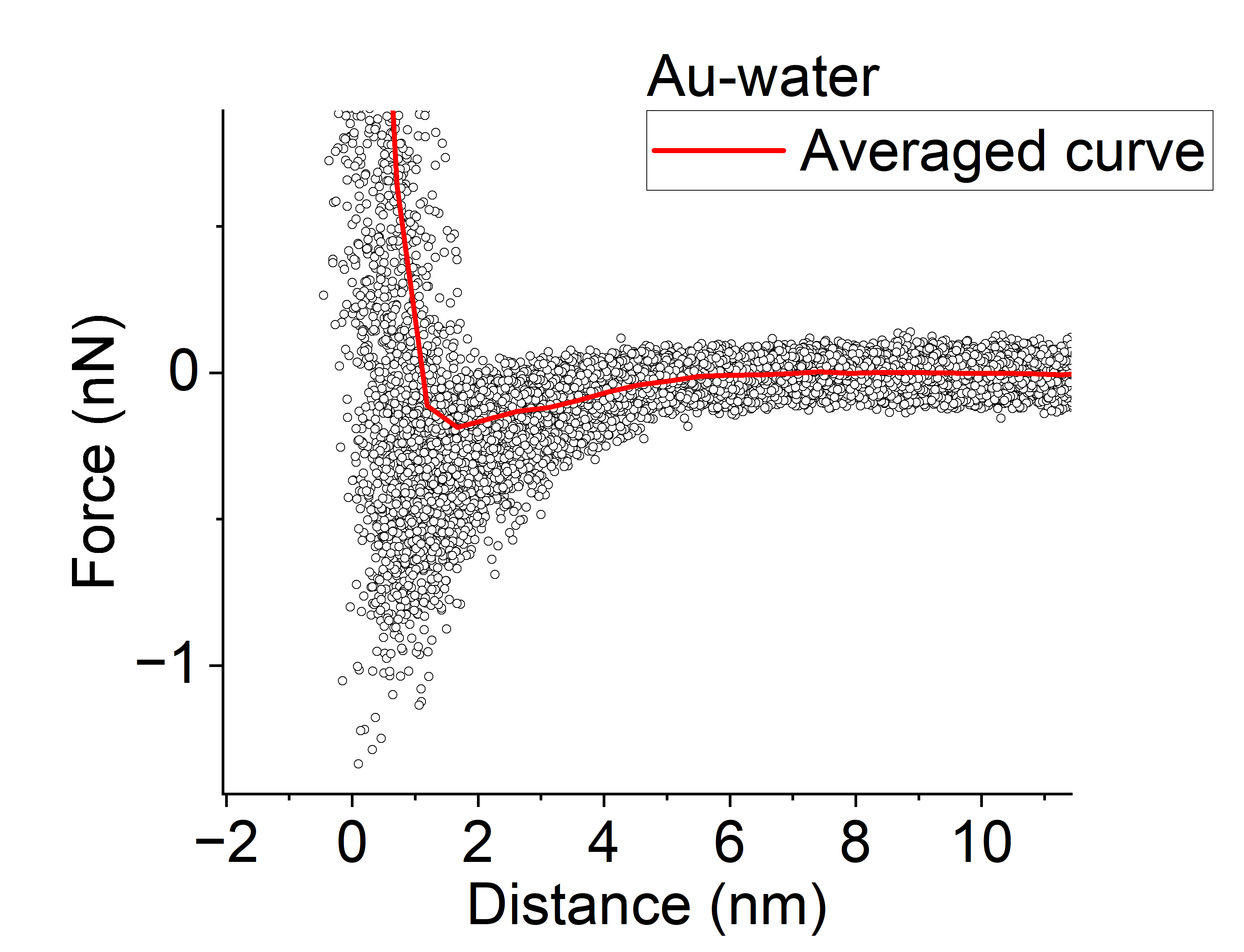 | **(F)**  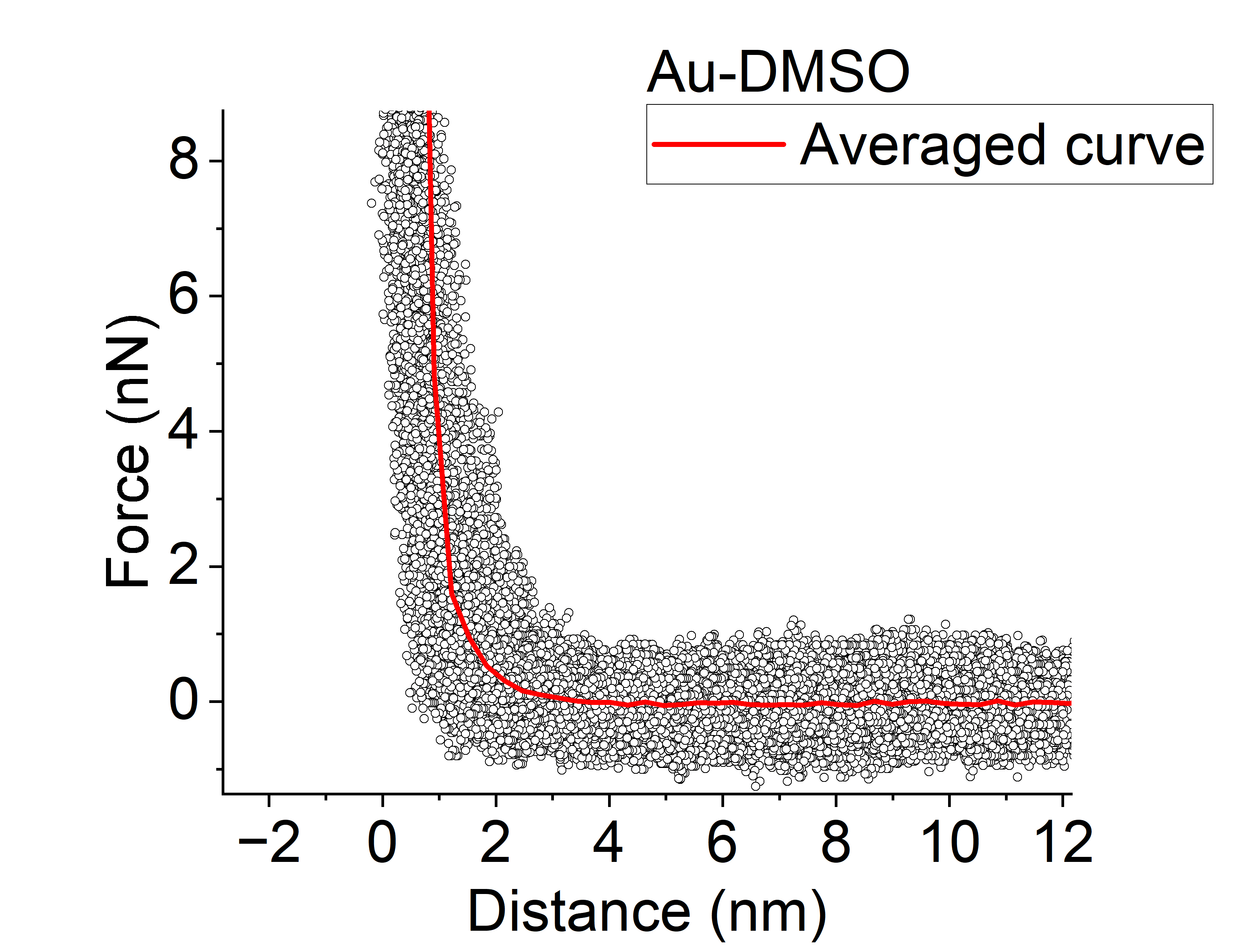 |

**Figure S2.** Raw data of representative force-distance curves (approaching segment) and averaged curve of symmetric system FOTT-coated Au across water (A), water:DMSO 3:1 (B), water:DMSO 2:1 (C), water:DMSO 1:1 molar ratio (D), and Au symmetric system across water (E), and DMSO (F). The experiments were conducted at room temperature (ca. 295 K).

# Section 3: contact angle measurements

| **(A)**  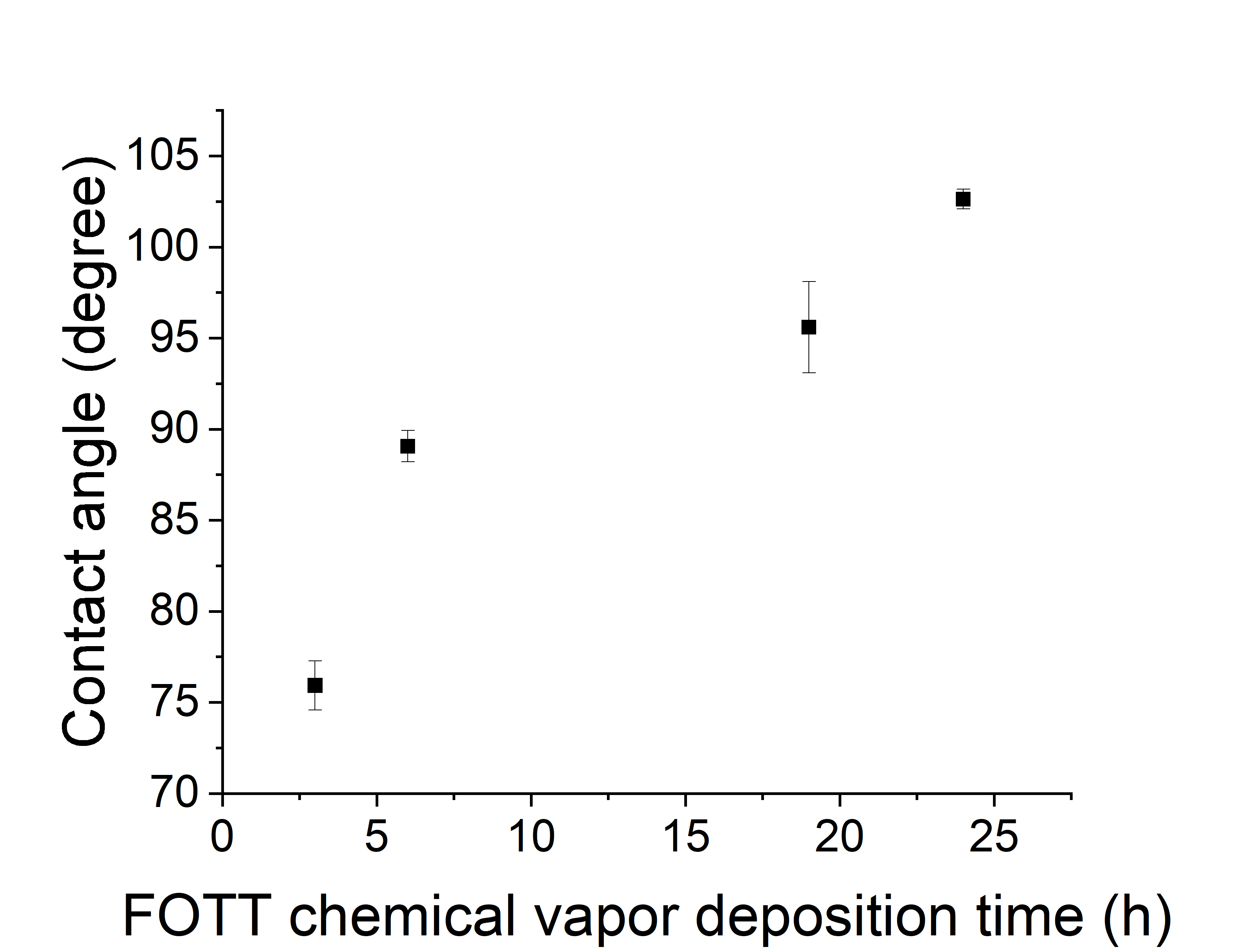 | **(B)**  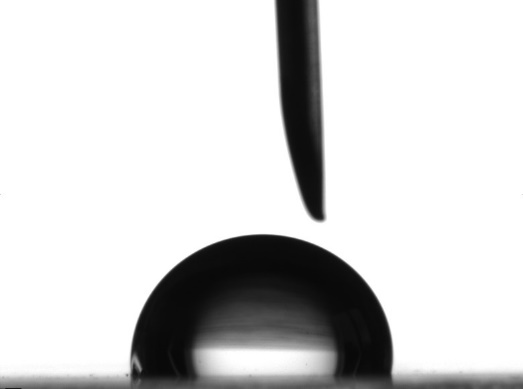 |
| --- | --- |
| **(C)**  **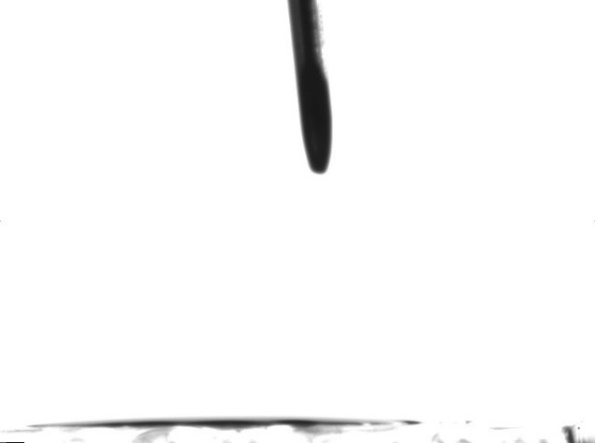** | **(D)**  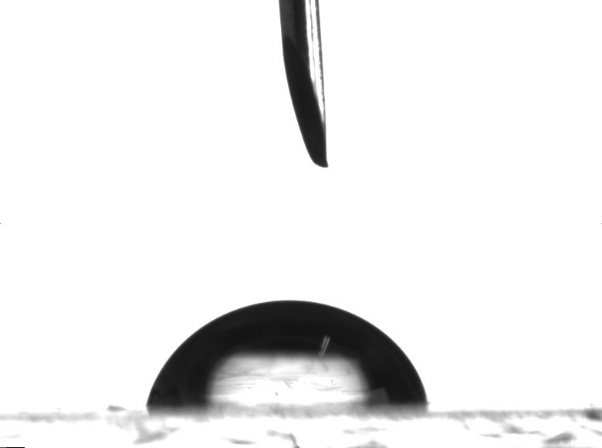 |

**Figure S3.** Time course studies of FOTT chemical vapor deposition on gold (A). Image of a 10 µL water drop on a FOTT-coated gold surface after 19 h of chemical vapor deposition (B), on a bare gold surface cleaned with ozone/UV for 1 h (C), and on a bare gold surface cleaned with ethanol (D). The experiments were conducted at room temperature (ca. 295 K).

# Section 4: Donaldson general potential for hydrophobic interactions

Donaldson and co-workers empirically derived an interaction potential when studying stressed surfactant bilayers (Donaldson et al., 2011). The initial expression was later generalized to account for hydrophobic and hydrophilic interfaces (Donaldson et al., 2013):

$W=-2\gamma\left( 1-\frac{a_{0}}{a} \right)exp\left( -\frac{D}{\lambda} \right)$ (S1)

Where W is the energy per unit area between interacting flat surfaces, γ is the interfacial energy, a is the area per molecule, a_0_ is the equilibrium area per molecule, D is the distance between surfaces and λ is the decay length. If we assume f=a_0_/a as the ratio of hydrophilic to hydrophobic area at a given interface, we obtain f=0 for a fully hydrophobic interface. Following, they introduced a more intuitive parameter, called Hydra parameter, H_y_, (i.e., H_y_=1- a_0_/a) to characterize the fraction of hydrophobic area at the interface. Hence, we can use the presented interaction potential for analysing AFM force-distance measurements between a particle and a plane, by using the Derjaguin approximation (Derjaguin et al., 1975):

$F=2\pi RW$ (S2)

Where F is the force and R is the radius of the AFM tip. Finally, if we rearrange Equation S1 and Equation S2, we obtain:

$\frac{F}{R}=-4\pi\gamma H_{y}exp\left( -\frac{D}{\lambda} \right)$ (S3)

# Section 5: force-distance curve simulation with multi-layer van der Waals model

Expressions for the force between two symmetric flat surfaces with adsorbed layers across a medium have been described in the literature (Parsegian 2005; Israelachvili 2011). However, experimental techniques such as atomic force microscopy normally comprise the use of a probe with a radius, R, and a flat sample of the same composition, interacting across a solvent. Thus, Equation E. S1 relates adsorbed-layer and bulk-substrate Hamaker constants, for a hydrocarbon-coated gold tip probing a hydrocarbon-coated gold flat surface across water (Stock et al., 2015):

$\frac{F}{R}=-\frac{1}{6}\left( \frac{A_{H232}}{D^{2}}-\frac{2\sqrt{A_{H232}A_{H131}}}{\left( D+T \right)^{2}}+\frac{A_{H131}}{\left( D+2T \right)^{2}} \right)$ (E. S1)

Where F is the force, R is the radius of the tip, D is the tip-sample distance, T is the thickness of the adsorbed monolayer, A_H232_ is the Hamaker constant of the adsorbed monolayers across water, and A_H131_ is the Hamaker constant of gold-gold across water. In our case A_H232_=6.7x10^-21^ J corresponds to FOTT-water-FOTT, which calculation is explained in the main text (see section 2.6), whereas A_H131_=7.8x10^-20^ J corresponds to gold-water-gold obtained fitting our experimental data with the van der Waals model (see vdW, Equation 1). Therefore, we performed simulations using Equation E. S1 to observe the influence of the thickness of the adsorbed monolayer in the interaction, as well as the magnitude of the bulk Hamaker constant. Hence, Figure S4A shows how the bulk material has little influence in the attractive interaction which is contrary to what we observed experimentally. Moreover, as the layer thickness increases, the range of interaction decreases. However, the substrate’s influence on the interaction becomes relevant if we increase the gold-water-gold Hamaker constant by one order of magnitude (Figure S4B).

| **(A)**   | **(B)**   |
| --- | --- |

**Figure S4.** Van der Waals (vdW) and multi-layer van der Waals simulated force-distance curves varying the thickness (T) of the adsorbed monolayer (A), and gold’s Hamaker (A_H131_) constant (B).

# Section 6: van der Waals retardation model fitting

Retarded van der Waals forces arise from the out of phase interaction between two bodies (Tabor and Winterton, 1968). This phenomenon is caused by the change of the instantaneous dipole of one molecule before it gets reached by the electric field generated by another polarized molecule (Butt et al., 2005). Note that retardation effects only affect the dispersion contribution and occur at larger distances than normal vdW forces (Israelachvili, 2011). Thus, for a tip with a radius, R, interacting with a flat surface, we used an extended vdW model, where the second term accounts for the retardation effect (Tabor and Winterton, 1968):

$F=-\frac{A_{H}R}{6D^{2}}-\frac{2\pi BR}{3D^{3}}$ (E. S2)

Where D is the tip-sample distance, A_H_ is the Hamaker constant, and B is the retardation constant. We used Equation E. S2 to fit the data. Thereby, Figure S5 discloses how the retardation model fits well in the gold-water symmetric system. Thus, we got a Hamaker constant value within the range of the one fitted with Equation 1 (see main text, section 3.1) and a retardation constant within the range (10^-28^ J m) of other reported systems (Israelachvili and Tabor, 1997; Stewart et al., 2002).

|  |
| --- |

**Figure S5.** Radius normalized force-distance approaching average curve between gold surfaces across water. The experiments were conducted at room temperature (ca. 295 K). The magenta line represents the vdW+retarded vdW model fit.

# Section 7: force-distance curves and their first derivatives

| **(A)**   | **(B)**   |
| --- | --- |
| **(C)**   |  |

**Figure S6.** Average force-distance approaching curves and corresponding 1st derivative of FOTT surfaces interacting across water:DMSO 3:1 (A), 2:1 (B), 1:1 (C) molar ratio mixtures. The values in red (i.e., 4.18, 3.95 and 3.40 nm) represent the distance at which the attractive decay starts. The experiments were conducted at room temperature (ca. 295 K).

# Section 8: control measurements

| **(A)**  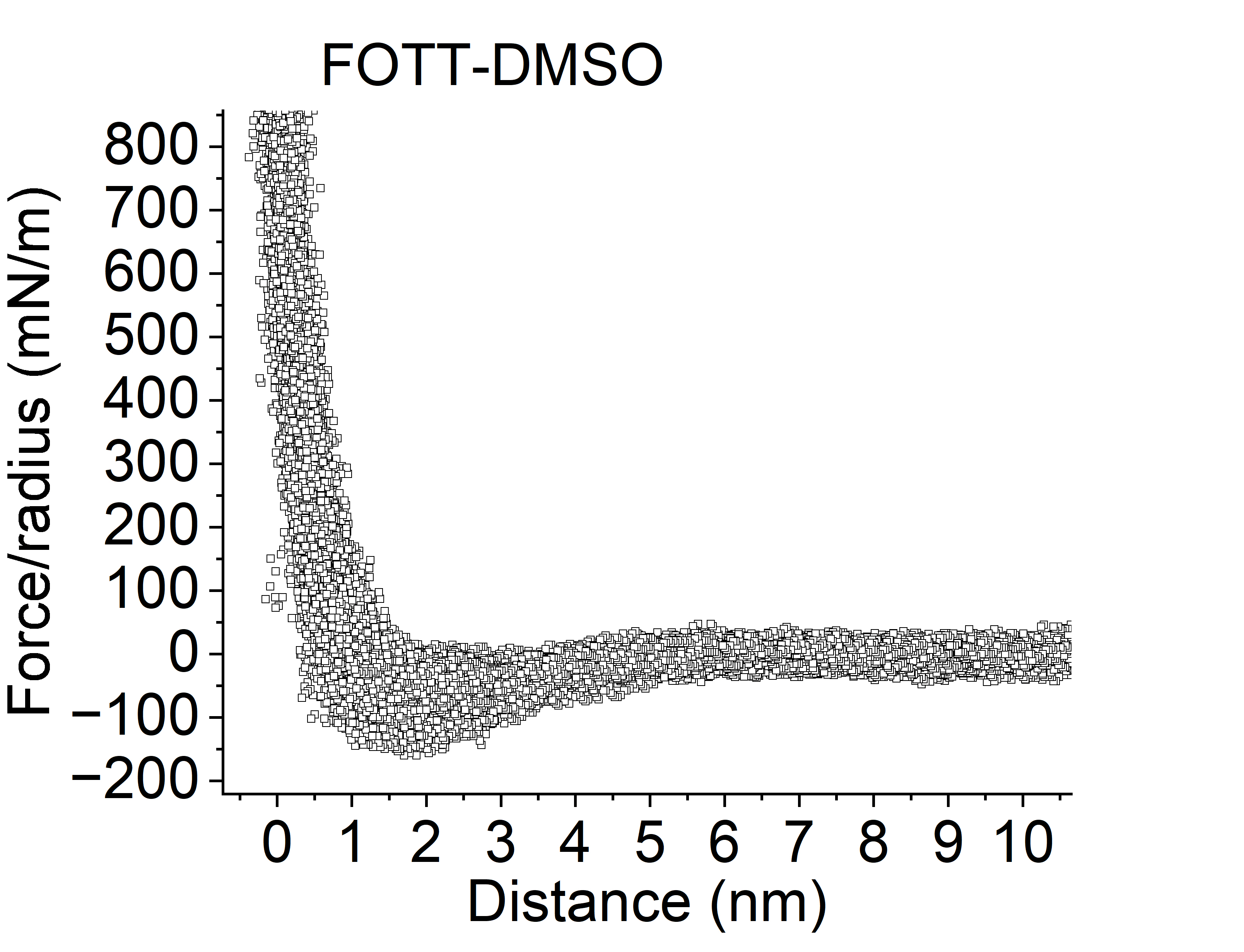 | **(B)**  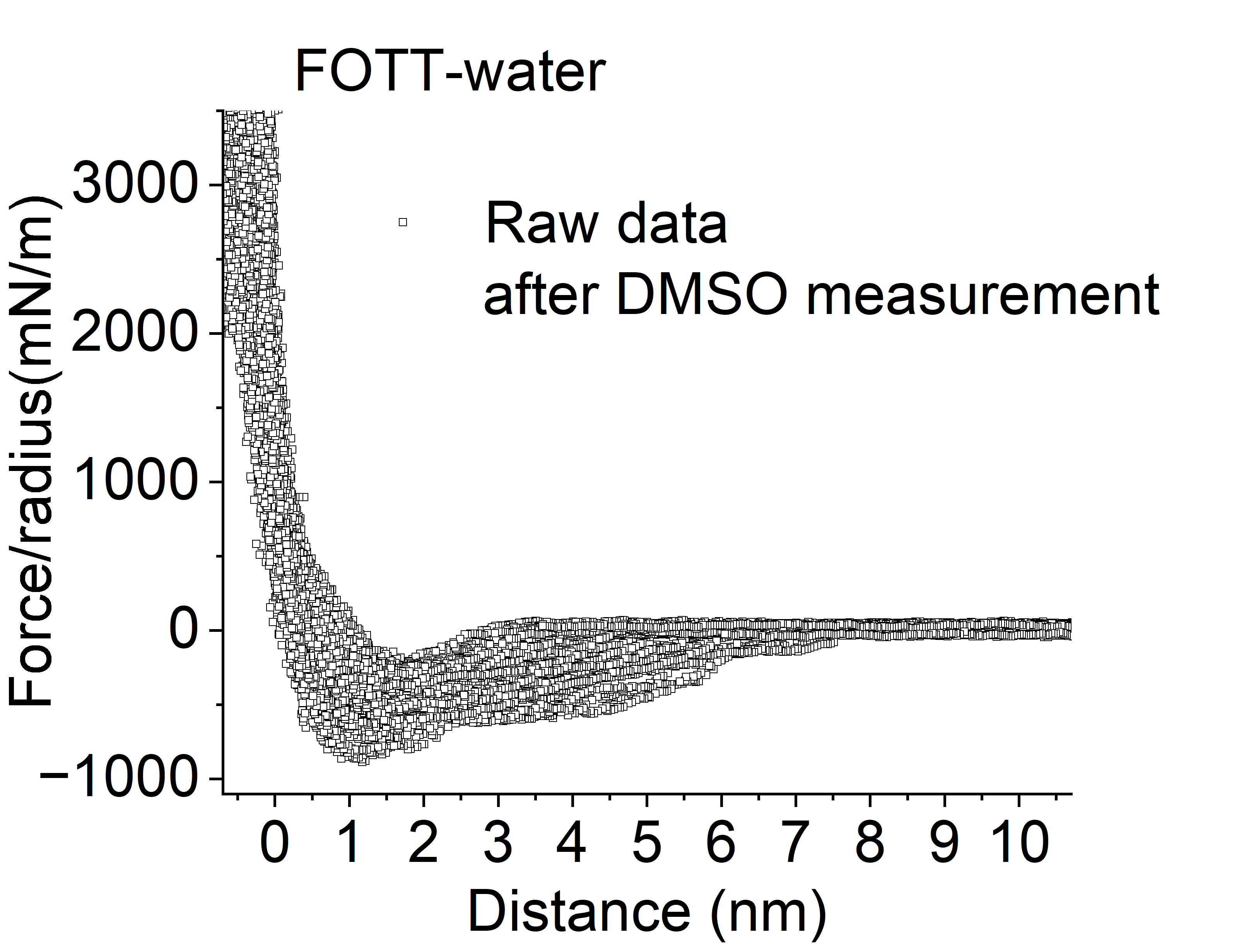 |
| --- | --- |

**Figure S7.** Raw data of radius normalized force-distance approaching curves of FOTT surfaces interacting across DMSO (A) and subsequent measurement across water (B). The experiments were carried out at room temperature (ca. 295 K).

**Figure S8.** Radius normalized average force-distance approaching curve replicas between FOTT surfaces across DMSO. The experiments were conducted room temperature (ca. 295 K).

| **(A)**  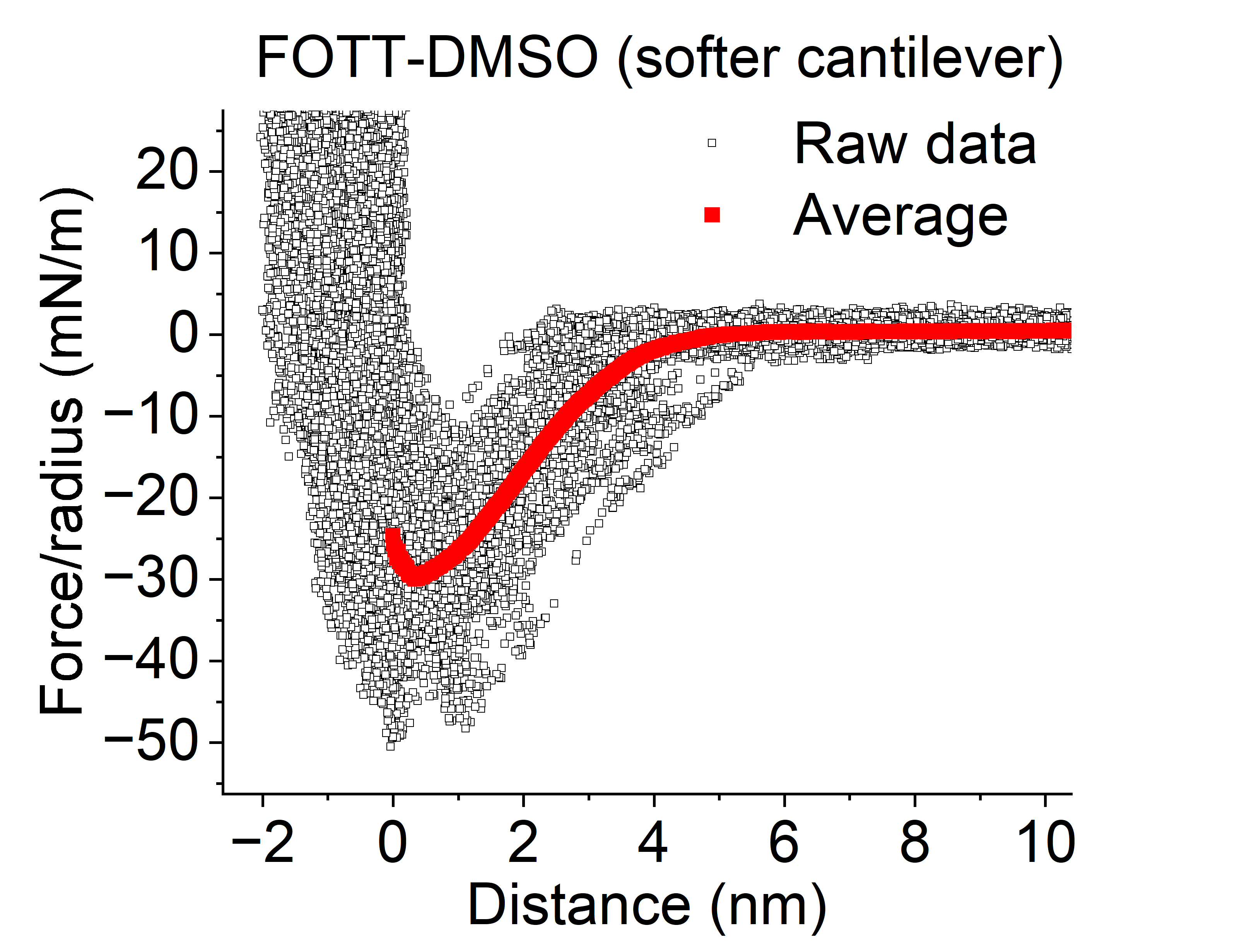 | **(B)**   |
| --- | --- |

**Figure S9.** Raw data and average of radius normalized force-distance approaching curves of FOTT surfaces interacting across DMSO measured with a soft cantilever (A), and fittings performed on the average curve (B). The blue line represents the extended van der Waals model fit, the magenta line represents the van der Waals model simulation. The experiments were conducted at room temperature (ca. 295 K).

**Figure S10.** Radius normalized average force-distance approaching curve between gold surfaces across DMSO. The shaded area represents the standard error. The experiments were conducted at room temperature (ca. 295 K).

# References

Butt, H.-J., Cappella, B., & Kappl, M. (2005). Force measurements with the atomic force microscope: Technique, interpretation and applications. Surface Science Reports, 59(1), 1–152. https://doi.org/10.1016/j.surfrep.2005.08.003

Derjaguin, B. v, Muller, V. M., & Toporov, Yu. P. (1975). Effect of contact deformations on the adhesion of particles. Journal of Colloid and Interface Science, 53(2), 314–326. https://doi.org/https://doi.org/10.1016/0021-9797(75)90018-1

Donaldson, S. H., Lee, C. T., Chmelka, B. F., & Israelachvili, J. N. (2011). General hydrophobic interaction potential for surfactant/lipid bilayers from direct force measurements between light-modulated bilayers. Proceedings of the National Academy of Sciences, 108(38), 15699–15704. https://doi.org/10.1073/pnas.1112411108

Donaldson, S. H. Jr., Das, S., Gebbie, M. A., Rapp, M., Jones, L. C., Roiter, Y., Koenig, P. H., Gizaw, Y., & Israelachvili, J. N. (2013). Asymmetric Electrostatic and Hydrophobic–Hydrophilic Interaction Forces between Mica Surfaces and Silicone Polymer Thin Films. ACS Nano, 7(11), 10094–10104. https://doi.org/10.1021/nn4050112

Israelachvili, J. N. (2011). Chapter 13 Van der Waals Forces between Particles and Surfaces. Intermolecular and Surface Forces (Third Edition, pp. 253–289). Academic Press. https://doi.org/10.1016/B978-0-12-391927-4.10013-1

Israelachvili, J. N., & Tabor, D. (1997). The measurement of van der Waals dispersion forces in the range 1.5 to 130 nm. Proceedings of the Royal Society of London. A. Mathematical and Physical Sciences, 331(1584), 19–38. https://doi.org/10.1098/rspa.1972.0162

Parsegian, V. A. (2005). Van der Waals forces: a handbook for biologists, chemists, engineers, and physicists. Cambridge University Press.

Stewart, A. M., Yaminsky, V. V., & Ohnishi, S. (2002). Measurement of Retarded Dispersion Forces of Mica. Langmuir, 18(5), 1453–1456. https://doi.org/10.1021/la0156311

Stock, P., Utzig, T., & Valtiner, M. (2015). Direct and quantitative AFM measurements of the concentration and temperature dependence of the hydrophobic force law at nanoscopic contacts. Journal of Colloid and Interface Science, 446, 244–251. https://doi.org/10.1016/j.jcis.2015.01.032
